# Cardiovascular Disease Association between Comorbidity and Cardiovascular and Cerebrovascular Events in Older Adults with NSTE-ACS

**DOI:** 10.1093/geroni/igaf122.2888

**Published:** 2025-12-31

**Authors:** Jingwen Shi, Ying Sun, Wen Tang, Yunli Xing, Qing Ma

**Affiliations:** Beijing Friendship Hospital, Capital Medical University, Beijing, Beijing, China (People’s Republic); Beijing Friendship Hospital, Capital Medical University, Beijing, Beijing, China (People’s Republic); Beijing Friendship Hospital, Capital Medical University, Beijing, Beijing, China (People’s Republic); Beijing Friendship Hospital, Capital Medical University, Beijing, Beijing, China (People’s Republic); Beijing Friendship Hospital, Capital Medical University, Beijing, Beijing, China (People’s Republic)

## Abstract

**Objective:**

To investigate the association between comorbidity and the occurrence of Major Adverse Cardiovascular and Cerebrovascular Events (MACCE) in older adults with Non-ST-Segment Elevation Acute Coronary Syndrome (NSTE-ACS) during one-year discharge. Materials and

**Methods:**

A total of 528 adults aged ≥ 65 years old were enrolled from January 2020 to November 2021 at Beijing Friendship Hospital, Capital Medical University for NSTE-ACS. The age-adjusted Charlson Comorbidity Index (aCCI) was calculated based on age and comorbidities. Participants were stratified into the low aCCI group (aCCI < 5) and the high aCCI group (aCCI ≥5). Multivariate COX regression analysis was employed to assess the association between aCCI and the occurrence of MACCE.

**Results:**

The high aCCI group included 251 patients (47.5%). This population exhibited advanced age, higher myocardial injury biomarker levels, and higher prevalence of frailty, polypharmacy, and reduced activities of daily living. When aCCI was analyzed as a continuous variable, the MACCE risk increased by 21.3% (adjusted HR = 1.213, 95%CI:1.043∼1.412, P = 0.012) as aCCI increased by one unit. When aCCI was analyzed as a categorical variable, it is exhibited that the MACCE risk increased by 85.8% (adjusted HR = 1.858, 95% CI: 1.119∼3.083, P = 0.017) in the high group compared to the low group.

**Conclusion:**

Higher comorbidity is associated with increased risk of MACCE during one-year discharge, suggesting that aCCI may serve as a predictor for adverse cerebrocardiovascular outcomes. Early assessment of comorbidity could conduct risk stratification in older adults with NSTE-ACS to implement health management for older adults.

